# First record of Harpellales, Orphellales (Kickxellomycotina) and Amoebidiales (Mesomycetozoea) from Bulgaria, including a new species of *Glotzia*

**DOI:** 10.3897/mycokeys.67.52055

**Published:** 2020-05-27

**Authors:** Laia Guàrdia Valle, Desislava Stoianova

**Affiliations:** 1 Unitat de Botànica, Dept. Biologia Animal, Biologia Vegetal i d’Ecologia. Fac. Biociènces. Universitat Autònoma de Barcelona. 08193-Bellaterra (Barcelona), Spain Universitat Autònoma de Barcelona Barcelona Spain; 2 Institute of Biodiversity and Ecosystem Research, Bulgarian Academy of Sciences. Sofia, Bulgaria Institute of Biodiversity and Ecosystem Research, Bulgarian Academy of Sciences Sofia Bulgaria

**Keywords:** aquatic insects, Balkans, gut fungi, symbiosis, trichomycetes, Zoopagomycotina

## Abstract

This paper presents the results obtained from a short survey performed in Bulgaria, southeast Europe, where the trichomycetes (sensu lato), an ecological group of arthropod gut endosymbionts, were previously completely unknown. The present study initiates the comprehension of these cryptic organisms, members of the Kickxellomycotina (Harpellales, Orphellales) and the Mesomycetozoea (Amoebidiales), in this Balkan country. Eighteen new geographic records for Bulgaria are reported, including 10 species of Harpellales, three species of Orphellales and five species of Amoebidiales. Within the Harpellales, the species *Glotzia balkanensis***sp. nov.** is described. This new species is most related to the rare species *G. centroptili* Gauthier ex Manier & Lichtw. and *G. stenospora* White & Lichtw., but is differentiated by spore and thallial characteristics. Photographs are provided and biogeographic implications of these records are discussed.

## Introduction

The ecological group trichomycetes includes filamentous or sac-like protozoan organisms (Mesomycetozoea Order Eccrinales and Amoebidiales) (Benny and O’Donnell 2000; Ustinova et al. 2000; Lutzoni et al. 2004; Tanabe et al. 2004; Adl et al. 2005; Cafaro 2005) and filamentous Fungi (Zoopagomycota, Kickxellomycotina, O. Harpellales and Asellariales) (Hibbet et al. 2007), all of them sharing the same ecological behaviour and living symbiotically inside the gut of arthropod hosts. The intense conditions and constrictions of this peculiar environment have shaped the convergent evolution, resulting in parallel morphology for all members in this ecological assemblage of organisms, explaining the previous classification of these phylogenetically unrelated orders within the Class Trichomycetes (Lichtwardt 1986). The Order Harpellales is the most diverse and well-known within the trichomycetes, with nearly 250 species, living associated mainly with immature stages of aquatic insects (Lichtwardt et al. 2001). Recently, a new order has been raised from within the Harpellales: the Orphellales L.G. Valle, M.M. White, Strongman & Lichtw to include the Plecopteran-associated genus, *Orphella*, with unusual characteristics in its sexual spores, amongst other particularities that make this genus exceptional within the Kickxellomycotina (White et al. 2018). The relationship between trichomycetes and their hosts is considered commensalistic, since they feed on the digestive content transiting those portions of the insect gut where most of the nutrients have already been absorbed by the animal (Misra 2001). However, they can behave mutualistically in some developmental and environmental conditions (Horn and Lichtwardt 1981), while other species may be deleterious to their hosts, like a few dipteran-associated species of *Smittium* which can be lethal to mosquitoes (Dubitskii 1978; Sweeney 1981; López-Lastra 1990) and may have an added interest for mosquito-control research.

Bulgaria is a biogeographically attractive region and, like most other Oriental European countries, it has not, until now, been studied by trichomycetologists. Europe transitions to Asia through the Balkans, acting as a connecting corridor, with Siberian and central European fauna and flora, together with Mediterranean components. This, combined with other geological factors, makes the Balkan Peninsula one of the two – together with the Iberian Peninsula– most interesting biogeographic regions in Europe, both being considered hotspots of biodiversity (Griffiths et al. 2004; Popov and Fet 2007). Bulgaria is actually the best-studied of all Balkan countries concerning biodiversity, because of the great tradition of zoological and botanical research (Popov and Fet 2007). With all these concurrent factors, it is our wish to contribute to the biodiversity data of the country with this preliminary study of arthropod endosymbionts.

At present, there are 54 species of trichomycetes documented from the Iberian Peninsula (Casas et al. 2019, Valle 2004, 2007, 2014a, 2014b, Valle and Santamaria 2002, 2004); it can be used as an indicator of what the diversity might be like in Bulgaria if more collections of trichomycetes are made in the future.

## Material and methods

All taxa reported here were collected from diverse localities (Table 1) in Bulgaria, most of them in the Provinces of Sofia and Pernik, since fresh material was processed in the laboratory of the Institute of Biodiversity and Ecosystem Research of the Bulgarian Academy of Sciences, in Sofia. Consequently, collecting trips where done not far away from the capital. Arthropod hosts, mainly aquatic insect larvae, nymphs and some aquatic isopods, were captured following the methods described by Lichtwardt et al. (2001), using aquatic dip nets and/or by hand-picking from stones, pebbles and vegetation. Hosts were transported to the lab in jars containing stream water on ice. Insect guts were dissected in water on microscope slides using a stereomicroscope and the gut symbionts transferred to a drop of water on another slide with the aid of ultrafine forceps and entomological needles. Lactophenol cotton-blue was used as the mounting medium for semi-permanent voucher slides, then these were sealed with clear fingernail polish. Most of the photographs were taken later at the Autonomous University of Barcelona (Catalonia, Spain) with a Zeiss Axioscope compound microscope, equipped with a Jenoptik ProgResC3 digital camera. For each of the endosymbiont species, the corresponding hosts were preserved in 70% ethanol for identification. Microscope slides are deposited in BCB-Mycotheca (herbarium at the institutional address of the corresponding author), except for some duplicates of the slides that were deposited in the Institute of Biodiversity and Ecosystem Research (Sofia, Bulgaria).

To reference the microscopic slides (specimens), a reference number was selected for each locality, preceded with the geographic reference BUL (Bulgaria: BUL–1, BUL–2 etc…). A second number was assigned sequentially for each microscope slide within the corresponding site (i.e. BUL–1–1: site 1, slide 1). See Table 1 for collecting sites details. All specimens collected by the authors (Leg.). All measurements, length × width in micrometres, for *Orphella* zygospores and diameter of the major outer spore coil × spore width (in micrometres) were made. Other measurements, as indicated in the text.

**Table 1.** Collection site information.

Ref	Province	Locality	EUNIS habitat type: name and code	Water Temp °C / pH	Geographic coordinates	Alt. (m a.s.l.)	Date (in 2016)
1	Sofia	Rakita River near Pasarel Village	Temporary running waters; C2.5	14°/7	42.547797N, 23.497202E	748	19 Aug
2	Sofia	Iskar River near Pasarel Village	Permanent non-tidal, smooth-flowing watercourses; C2.3	11.5°/7.2	42.535885N, 23.508824E	712	19 Aug
3	Sofia	Small creek next to Iskar	Non-permanent temporary	12°/7	Close to the previous locality	710	19 Aug
4	Pernik	Small brook tributary of Struma River, Chuypetlovo Village	Permanent non-tidal, fast, turbulent watercourses; C2.2	12°/6.2	42.520650N, 23.245939E	1258	20 Aug
5	Pernik	Struma River, Chuypetlovo Village	Permanent non-tidal, fast, turbulent watercourses; C2.2	11°/6.2	42.520608N, 23.245650E	1255	20 Aug
6	Pernik	Small brook tributary of Struma River after Bosnek Village near the bridge	Permanent non-tidal, smooth-flowing watercourses; C2.3	18°/7	42.494986N, 23.171681E	909	20 Aug
7	Sofia	Tributary of Chureshka River, near Eleshnitsa Village	Permanent non-tidal, fast, turbulent watercourses; C2.2	14°/6.8	42.760899N, 23.648960E	707	21 Aug
8	Sofia	Chureshka River, bridge before Potop Village	Permanent non-tidal, fast, turbulent watercourses; C2.2	14.5°/6.8	42.752736N, 23.647861E	669	21 Aug
9	Sofia	Small pond (swamp) near highway Hemus	Permanent eutrophic lakes, ponds and pools; C1.4/ C1.3	18.7°/6.2	42.773999N, 23.774886E	903	21 Aug
10	Kyustendil	Manastirska River before Rilski Manastir	Permanent non-tidal, fast, turbulent watercourses; C2.2	9°/6.1	42.153581N, 23.389001E	1422	22 Aug
11	Kyustendil	Rilska River before Pastra Village	Permanent non-tidal, fast, turbulent watercourses; C2.2	10.5°/6.1	42.113840N, 23.318704E	1027	22 Aug
12	Sofia	Darvenishka River, Sofia city, Park Vertopo	Permanent non-tidal, smooth-flowing watercourses; C2.3	18°/7.2	42.645710N, 23.364568E	585	23 Aug

## Results

### Order Harpellales

#### Genistellospora homothallica

Taxon classificationFungiHarpellalesLegeriomycetaceae

Lichtw, 1972.

C4105CDC-6BAA-5F51-A3DE-1F1CD492D6F6

Figs 1 , 2

##### Specimens examined.

Site 2: slides BUL–2–1, BUL–2–6, BUL-2-7 (zygo.), BUL–2–10; Site 3: slides BUL–3–1; site 4: slides BUL–4–10; Site 7: slides BUL–7–1, BUL–7–2, BUL–7–3; site 8: slides BUL–8–3; site 12: slide BUL–12–5.

##### Notes.

*Genistellospora homothallica* is a cosmopolitan species and its Simuliidae hosts are widespread and common in varied environments (Lichtwardt et al. 2001), especially in fast flowing waters. This species has been previously documented from many countries in the Northern Hemisphere, including USA (Lichtwardt 1972), Canada (Moss and Lichtwardt 1976), United Kingdom (Lichtwardt 1986), Spain (Santamaria and Girbal 1998), France (Valle 2004), Italy (Valle et al. 2013) and Portugal (Valle 2013a). The species has also been recorded from Southern tropical regions, including Costa Rica (Lichtwardt 1997), Puerto Rico (White et al. 2000), Argentina (López-Lastra et al. 2005), Dominican Republic (Valle and Cafaro 2010), Chile (Lichtwardt and Arenas 1996) and Colombia (Barón and Valle 2018). Trichospores of *G. homothallica* are typically ovate-elongated, slightly asymmetrical, measuring 34–40 × 10.5–12 μm in our collections. Young zygospores were observed in one Bulgarian specimen (BUL–2–7) with the characteristic zygosporophore of the species, bearing a straight or reflexed thumb-like terminal cell measuring 43–58 μm length. Often, *G. homothallica* thalli were covered with thalli of the epithallic *Simuliomyces microsporus* Lichtw., as seen in Fig. 2 (arrows).

**Figures 1, 2.**
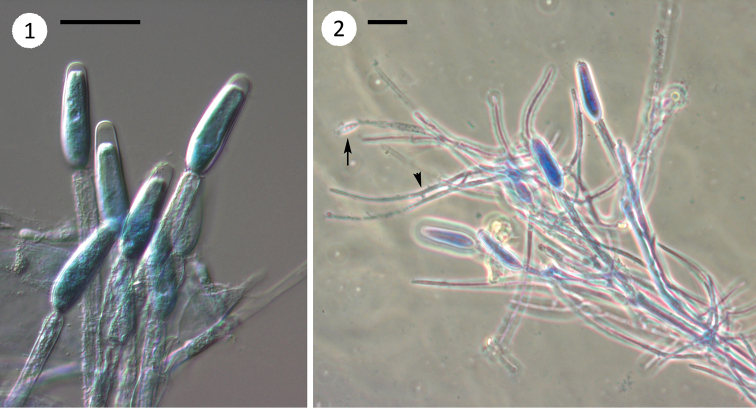
*Genistellospora homothallica* and *Simuliomyces microsporus* from Simuliidae larvae. **1** Fertile branches of *G. homothallica* with terminal trichospores **2** fertile branches and trichospores of *G. homothallica* with an attached thallus of *S. microsporus* showing a trichospore (arrow) and conjugation tubes (arrowhead). Scale bars: 25 μm in all figures.

#### Glotzia balkanensis

Taxon classificationFungiHarpellalesLegeriomycetaceae

LG Valle & D Stoianova sp. nov.

64AB4B7E-3592-54F4-9967-13F13A1E4A06

834966

Figs 3–10

##### Holotype.

Bulgaria, Sofia, Pasarel Village, Iskar River, 42.535885N, 23.508824E; 712 m a.s.l.; 19 Aug 2016; LG Valle and D Stoianova Leg; In the hindgut of *Baetis melanonyx* Pictet (Baetidae, Ephemeroptera); microscope slide BCB–BUL–2–2.

##### Paratypes.

Same locality and date as the holotype; microscope slide BUL–2–3, BCB–BUL–2–4. Bulgaria, Sofia capital city, Darvenishka River, Park Vartopo, 42.645710N, 23.364568E; 585 m a.s.l.; 23 Aug 2016; LG Valle and D Stoianova Leg; In the hindgut of *Baetis melanonyx* (Baetidae, Ephemeroptera); microscope slide BCB-BUL–12–3.

##### Etymology.

*Balkanensis*, from the Balkan Peninsula.

##### Description.

**Figures 3–10.**
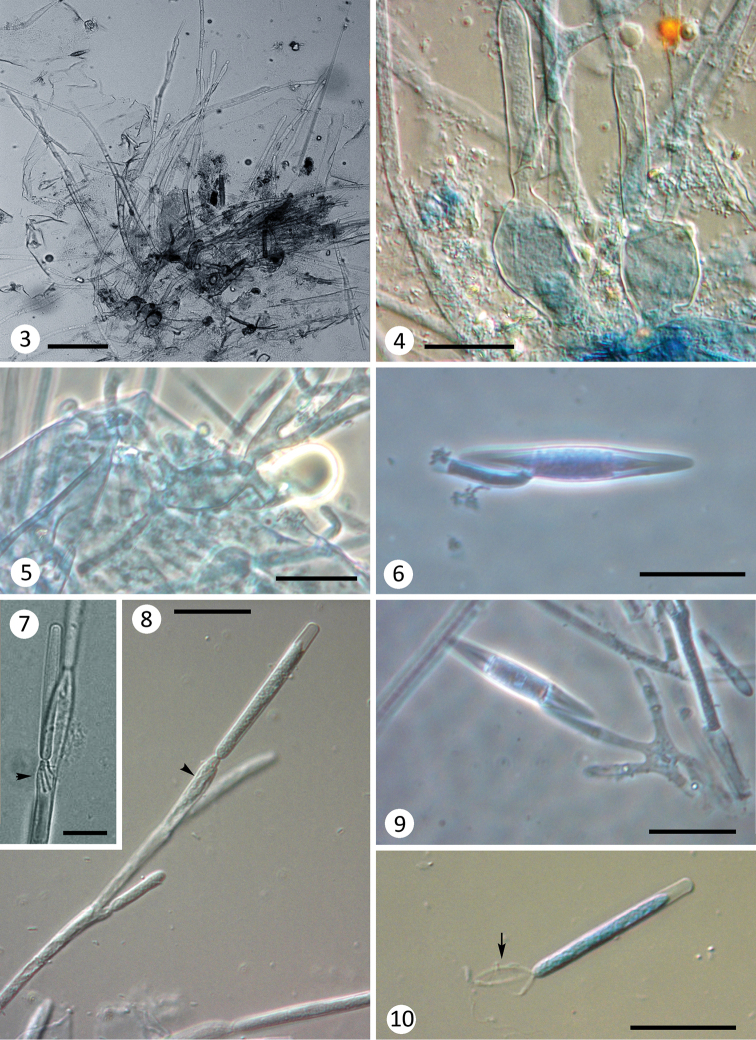
*Glotzia balkanensis* sp. nov. from Baetidae nymphs. **3** thallus overview, note the inflated and branched basal cell structures **4, 5** detail of various swollen basal cells **6** loose zygospore with a collar **7, 8** trichospores on fertile branches, see the appendages inside the generative cell (arrowheads) **9** Zygospore arising from a conjugation tube **10** loose trichospore with a central long filiform appendage and two smaller lateral appendages. Scale bars: 50 µm (**3**), 25 µm (**4–10**).

Thalli measuring up to 600 μm long. Basal cell broadly inflated (18–30 μm diam.) and often branched (Fig. 3), bearing a small discoid secreted holdfast at the base or laterally to the basal cell axis (Fig. 4). Dichotomous branching above the basal cell; distal branches bearing spores (Figs 2, 8). Trichospores cylindrical, with a terminal refractive cap (not always visible), measuring 44–56 × 4.5–5.5 μm, with 3 appendages, one central long filiform appendage, coiled around two shorter (about 15–20 μm) and broader lateral appendages (Fig. 10). These appendages can be seen within the generative cell while still attached (Figs 7, 8 arrowhead). Fertile branches bearing 3–4 generative cells, measuring 20–35 × 4–6 μm. Zygospores biconical, 48–60 × 7.5–9.5 μm, with a collar 5–10 (–16) × 4 μm, attached eccentrically and laterally (Type II) to a zygosporophore 20–30 μm long, arising from the conjugation tube in series of scalariform conjugations (Fig. 9). In the hindgut of Baetidae (Ephemeroptera) nymphs.

##### Notes.

The genus *Glotzia* has nine species (including that described here), all of them sharing the characteristic cylindrical trichospores with a slightly globose cap and the peculiar 3-appendage arrangement observed also in *G. balkanensis*. This new species mostly resembles the type species *G. centroptili* described by Gauthier (1936) in French pools and streams of Dauphiné province (south-eastern France) from *Centroptilum luteolum* nymphs (Baetidae). This French species was recently rediscovered in Catalonia (Spain) also within *Centroptilum* sp. nymphs (Busquets et al. 2018). This second observation in Spain was important for providing new material to complete the original description, which was scant and had no photographs, only a drawing of a single specimen (Gauthier 1936). The specimens from Bulgaria can be differentiated from *G. centroptili* by spore characteristics. Trichospores of the Bulgarian species are longer than those observed in France or Spain (40 × 4 μm according to Gauthier (1936); 35–43 × 4–6 μm according to Busquets et al. (2018), up to 56 μm in the specimens reported here). All the fertile branches observed had a maximum of four generative cells in *G. balkanensis*, while up to seven have been reported in *G. centroptili*. Zygospores of *G. balkanensis* are quite similar to those of *G. centroptili* in length, but they have significantly larger diameter in the French species, 15 μm diameter (according to Gauthier 1936), while only 7.5–9.5 μm (8.4 μm average) in *G. balkanensis*. Unfortunately, the specimens of *Glotzia centroptili* collected from Spain, had no zygospores to compare with the new species, only trichospores were observed and, thus, we do not have a broad perspective of the zygosporic variation in this species, because apparently, the description of the type species was based on just a few specimens (Busquets et al. 2018). The presence of a collar on released zygospores was not described by Gauthier in *G. centroptili*. The species described here has a quite variable collar length, but in most zygospores, it is rather short (5–10 μm). Regarding thallial characteristics, both species are quite similar, but there are major differences in their fertile branches, generative cells and in the basal cell. The basal cell is much more swollen in the Bulgarian species, resembling (but not identical to) that of the Italian species *Glotzia distorta* LG Valle, Santam. & W Rossi which has different spore features (Valle et al. 2014). Most species of *Glotzia* are associated with Baetidae (Ephemeroptera), except for one species recorded in a New Zealand Plecoptera nymph, *Glotzia plecopterorum* Lichtw. (Williams and Lichtwardt 1990) and another species living within Dixidae (Diptera) larvae *Glotzia incilis* Strongman & MM White, (Strongman and White 2008). Actually, *Glotzia* is one of the Harpellid genera with a wider host range. *Glotzia centroptili* was recorded from *Centroptilum* (Baetidae) in France and Spain and *G. distorta* from the related *Procleon pennulatum* (= *Centroptilum pennulatum*) (Valle et al. 2014). However, *Glotzia balkanensis* has been recorded from a different host, *Baetis melanonyx*, but in the same family Baetidae. In fact, this is the first record of a Harpellid fungus within this host species.

#### Graminella bulbosa

Taxon classificationFungiHarpellalesLegeriomycetaceae

Léger & Gauthier, 1937 ex Manier, 1962.

78D22DC9-A2A8-5535-A187-40E3A3D5CD0E

Figs 11–13

##### Specimens examined.

Site 2: slide BUL–2–5; Site 8: slides BUL–8–1, BUL–8–2.

##### Notes.

**Figures 11–16.**
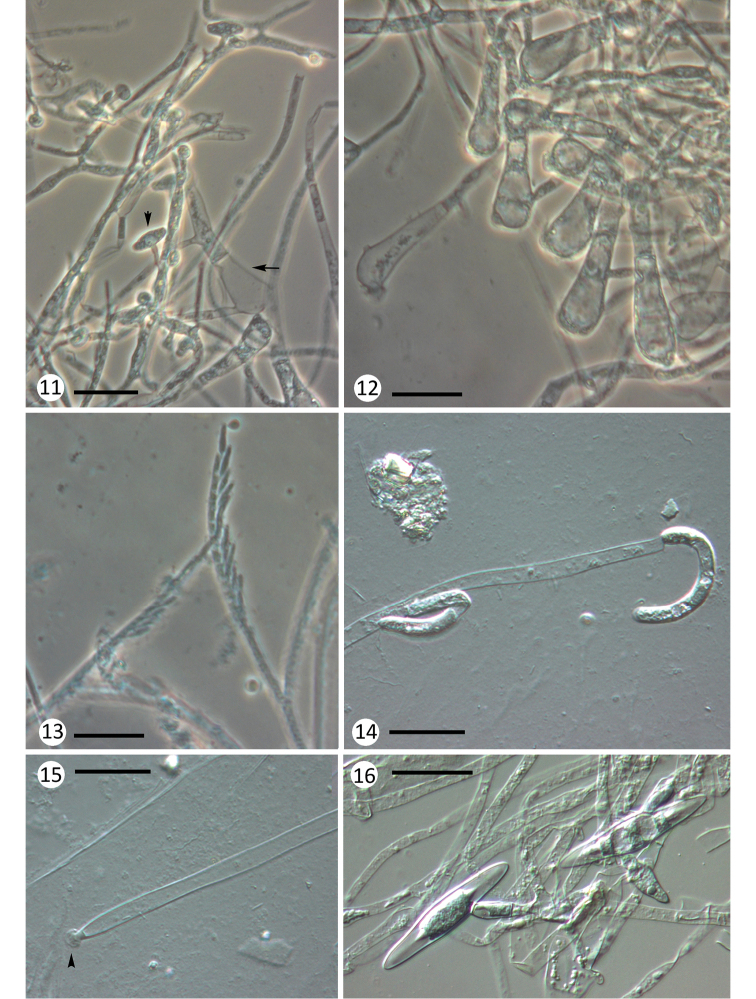
Various species of Harpellales. **11–13***Graminella microspora* from Baetidae nymphs: **11** thallus overview, with inflated basal cell structures from which propagules arise or are extruded (note: one empty basal cell which has extruded its cellular content, arrow); young zygospores can also be observed (arrowhead) **12** a cluster of basal cells **13** fertile branch with a long series of minute trichospores; **14, 15***Harpella melusinae* from Simuliidae larvae: **14** generative cells and allantoids or curved trichospores **15** basal cell and small conic holdfast **16***Legeriomyces ramosus* from Baetidae nymphs, zygospores and zygosporophores. Scale bars: 25 μm in all figures.

This species is characterised by the unusual formation of vegetative propagules from the bulbous basal cells (Fig. 12), a feature only shared with the related genus *Gauthieromyces* (Lichtwardt 1983). *Graminella bulbosa* was described from France (Léger and Gauthier 1937; Manier 1962). The species is also known from Spain (Valle 2007), Portugal (Valle 2013a) and Italy (Valle et al. 2013). *Graminella bulbosa* has been reported associated with various species of *Baetis* and related genera, very frequently within the hindgut of *B. rhodani* (Pictet). This species of mayfly is common and widespread in Europe and it also hosted Bulgarian specimens of *G. bulbosa* in the surveyed rivers, together with *B. alpinus* (Pictet). In fact, the genus *Baetis* bears different Harpellid species, including the more common *Legeriomyces ramosus*, occasionally sharing the same gut lumen with *Graminella bulbosa*. Bulgarian specimens of *G. bulbosa* show the typical small and numerous trichospores (Fig. 13), measuring 8 –11 × 2 µm in our collections. These measurements are midway between *G. bulbosa* and *G. microsporus* (see discussion for further information). Unfortunately, only immature zygospores were observed (Fig. 11 arrowhead).

#### Harpella melusinae

Taxon classificationFungiHarpellalesLegeriomycetaceae

Léger & Duboscq, 1929.

42ED13A4-3ACF-5739-B17B-9CE300384F04

Figs 14–15

##### Specimens examined.

Site 2: slides BUL–2–1, BUL–2–6, BUL–2–7, BUL–2–10; site 4: slides BUL–4–10; Site 7: slides BUL–7–1, BUL–7–2, BUL–7–3; Site 8: slides BUL–8–3; site 12 slide: BUL–12–5

##### Notes.

A cosmopolitan or sub-cosmopolitan species, widespread in the Northern Hemisphere, it is common in European localities where their hosts were available. It has been found attached to the peritrophic matrix of Simuliidae larvae in different Bulgarian localities. Our specimens have the typical characteristics of the species, distinguishable on the basis of trichospore morphometry (Fig. 14) and holdfast structure (Fig. 15) (Léger and Duboscq 1929a). The trichospores in our specimens measured 50–60 × 6–7 μm and were variable in shape, from nearly straight to allantoid or slightly coiled.

#### Legeriomyces ramosus

Taxon classificationFungiHarpellalesLegeriomycetaceae

Pouzar, 1972.

B7616663-068F-52A5-A816-4D1C1DF34F9E

Fig. 16

##### Specimens examined.

Site 2: slide BUL–2–9; Site 11: slides BUL–11–1 (zygo.), BUL–11–2, BUL–11–3.

##### Notes.

This species was found on the hindgut lining of Baetidae hosts (*Baetis rhodani* Pictet, *Baetis melanonyx* (Pictet) and *B. alpinus* (Pictet). The species seems to have a cosmopolitan distribution, at least in the Northern Hemisphere (Lichtwardt et al. 2001). In Europe, the species is common, with records from France, where it was originally described under the name of *Genistella ramosa* (Léger and Gauthier 1932) (Pouzar 1972), from United Kingdom (Moss 1979), Switzerland (Lichtwardt 1986), Spain (Valle and Santamaria 2002), Norway (White and Lichtwardt 2004), Sweden (Lichtwardt 1984), Portugal (Valle 2013a) and Italy (Valle et al. 2013). *Legeriomyces ramosus* has been reported also from India (Misra and Tiwari 2008), China (Strongman et al. 2010), from several localities in USA (Lichtwardt 1986) and Canada (Strongman 2010). Bulgarian specimens have, as is usual in this species, a broad range of trichospore variability, measuring 30–40 ×7–8.5 µm, with two appendages differing in length. Zygospores measure 50–61 × 9–12 µm in our collections (Fig. 16).

#### Simuliomyces microsporus

Taxon classificationFungiHarpellalesLegeriomycetaceae

Lichtw, 1972.

682AC306-054C-57A9-8E93-274A3A33507D

Fig. 2

##### Specimens examined.

site 2: slides BUL–2–1, BUL–2–6, BUL–2–10; site 3: slides BUL–3–1; Site 7: slide BUL–7–3(zygo.); Site 12: slide BUL–12–5.

##### Note.

This species was obtained from the hindgut lining of Simuliidae larvae. Most of the observed specimens of *S. microsporus* were attached to thalli of both *Genistellospora homothallica* (Fig. 2) and *Paramoebidium chattoni*. All the individuals had trichospores and one of the specimens showed also the typical type-I zygospores’ young (Fig. 2). All characteristics and measurements of Bulgarian *S. microsporus* match that of previous descriptions of the species in Europe (Moss 1970; Valle 2004, White and Lichtwardt 2004). This species, discovered in the USA (Lichtwardt 1972), has a sub-cosmopolitan distribution (see Lichtwardt et al. 2001), with a patchy distribution in the Northern Hemisphere and also Australia (Lichtwardt and Williams 1992).

#### Smittium dipterorum

Taxon classificationFungiHarpellalesLegeriomycetaceae

Lichtw., 1997.

1D88A282-CD24-5934-A131-931896105AD0

Fig. 17

##### Specimen examined.

site 12: slides BUL–12-1; BUL–12-4.

##### Notes.

**Figures 17–20.**
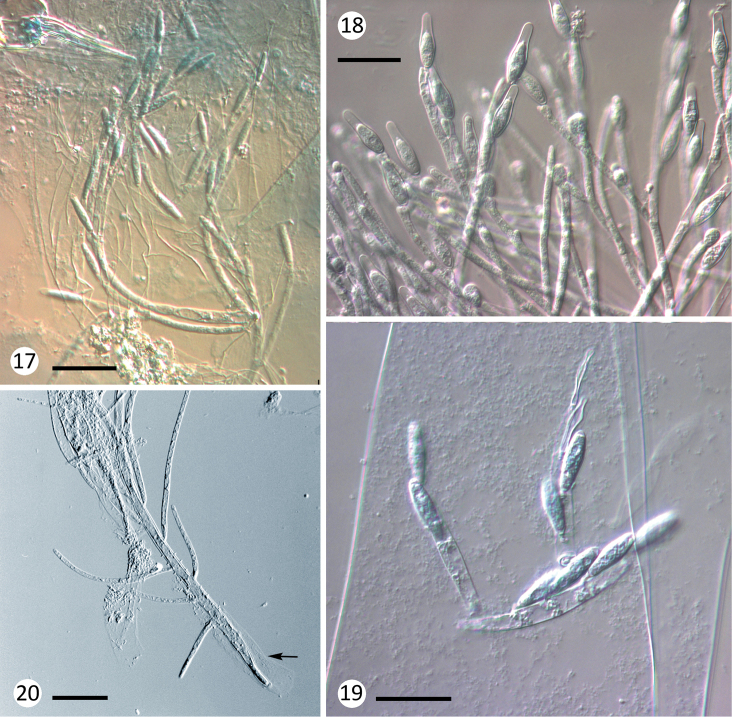
Various species of Harpellales. **17***Smittium dipterorum* from Chironomidae larvae; thallus overview with fertile branches and trichospores **18***Spartiella barbata* from Baetidae nymphs, fertile branches with trichospores **19***Stachylina nana* from Chironomidae larvae, thallus overview with trichospores **20***Stipella vigilans* from Simuliidae larvae, thallus with mucilaginous material at the basal cell (arrow). Scale bars: 25 μm in all figures.

This species was previously known from Costa Rica (Lichtwardt1997), Spain (Valle and Santamaria 2004), Dominican Republic (Valle and Cafaro, 2010) and Mexico (Valle et al. 2011) in the tract of Simuliidae and Chironomidae. Bulgarian specimens were associated with Chironomidae midges (*Chironomus* sp.). They had cylindrical-elongated trichospores measuring 16–19 × 3–4 µm, with a short collar about 2.5 µm, slightly flared outwards. The thallus is branched at the base, with verticillate apical branching (Fig. 17). Each fertile branch has 4–6 generative cells. Specimens from Bulgaria resemble most of those described from Spain.

#### Spartiella barbata

Taxon classificationFungiHarpellalesLegeriomycetaceae

Tuzet & Manier ex Manier, 1968.

E1887378-A182-50D0-8C75-6A367054D737

Fig. 18

##### Specimens examined.

Site 7: slide BUL–7–4; Site 10: slide BUL–10–3.

##### Notes.

This species was described from France (Tuzet and Manier 1950) in the hindgut of Baetidae nymphs. *Spartiella barbata* is distinguished by its lobulate basal cell, obpyriform trichospores, measuring 21–26.5 × 6.5–7.5 µm in our specimens (Fig. 18) and the presence of one appendage tightly coiled just after release and then eventually uncoiling into a long delicate filiform structure. *Spartiella barbata* seems to be more common in Europe, where it has been recorded from France (Tuzet and Manier 1950, Manier 1962b), United Kingdom (Lichtwardt 1986) and Spain (Valle 2007), with only one report of the species from North America, in Canada (Strongman 2010).

#### Stachylina nana

Taxon classificationFungiHarpellalesLegeriomycetaceae

Lichtw., 1984.

C55784E6-C5F7-5195-86FC-B6642C1CC67A

Fig. 19

##### Specimens examined.

Site 11: slide BUL–11–4.

##### Notes.

*Stachylina nana* was found in the mid-gut of Chironomidae (*Chironomus* sp.) in the same host as *Smittium dipterorum*. Specimens of *S. nana* in Bulgaria had a small thallus, 60–70 × 7–8 µm, with 1–4(-5) generative cells. Trichospores measure 20–24 × 7–7.5 µm, without a collar. All measurements agree with the original description of the species (Lichtwardt 1984). This species was recorded before from Europe, including France (Lichtwardt 1984) and Spain (Santamaria and Girbal 1997); Asia, in Thailand (Hapsari et al. 2009), China (Strongman and Wang 2015) and also America, including Canada (Strongman 2007, 2010; Strongman and White 2008) and USA (Beach and White 2012). Probably, *S. nana* has a cosmopolitan distribution, although we only have patchy data from few surveyed countries.

#### Stipella vigilans

Taxon classificationFungiHarpellalesLegeriomycetaceae

Léger & Gauthier, 1932.

BC2FE655-9A63-5C19-99FB-6AE44A9CE22C

Fig. 20

##### Specimens examined.

Site 4: slides BUL–4–3, BUL–4–11.

##### Notes.

*Stipella vigilans* was originally described from the French Alps in the hindgut of Simuliidae, together with the protozoan *Paramoebidium* sp. (Léger and Gauthier 1932). This species was also reported from Spain (Valle 2007), England (Moss 1970), Armenia (Nelder et al. 2005), Thailand (Hapsari et al. 2009). The species is easily distinguished by the particular basal cell, simple or forked, verrucose and narrowing in the most basal section, attached to the hindgut by means of a mucilaginous adhesive substance (holdfast) (Fig. 20). The trichospores of *S. vigilans* are also very characteristic, almost cylindrical, measuring 35–50 × 3–4.5 µm in our collections, although probably somewhat young, they fit the original description, according to Léger and Gauthier (1932). We did not see released trichospores, since none of them was mature enough in our collections. Trichospores in this species bear three petaloid appendages, which are visible inside the generative cells before detachment.

### Order Orphellales

#### Orphella catalaunica

Taxon classificationFungiOrphellalesLegeriomycetaceae

Santam & Girbal, 1998.

2B182C9D-0440-54F8-8FE1-52D0688C8B08

Fig. 21

##### Specimens examined.

Site 4: slides BUL–4–1, BUL–4–5, BUL–4–7; site 7: slide BUL–7–6.

##### Notes.

**Figures 21–25.**
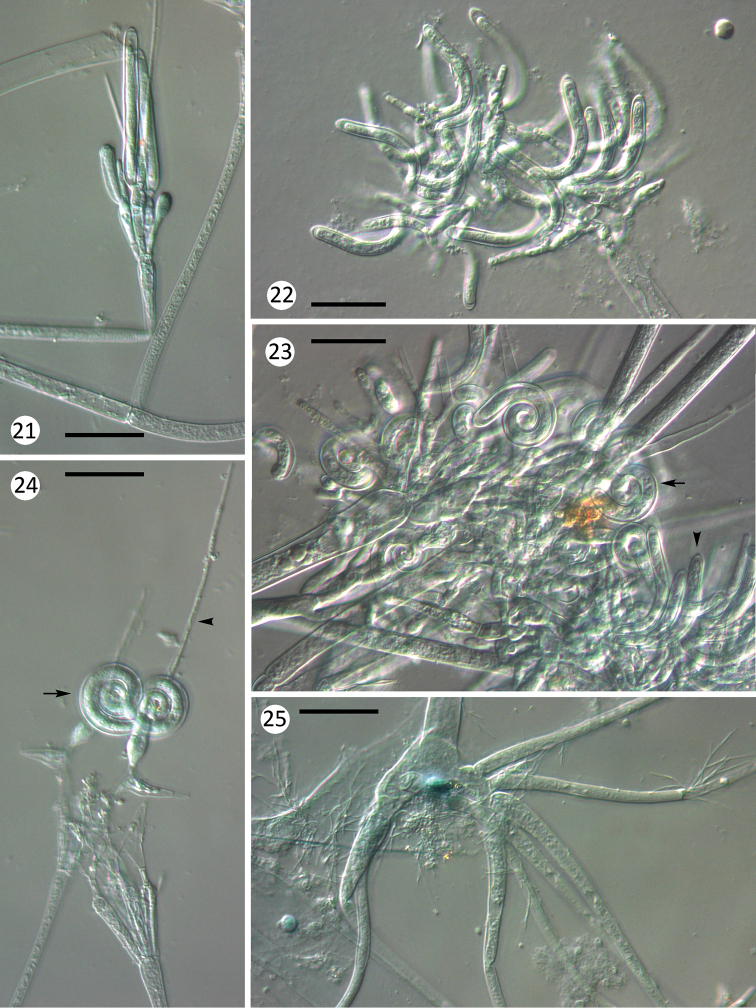
Various species of Orphellales. **21***Orphella catalaunica* from Leuctridae nymphs, trichospores and accompanying cells **22, 23***Orphella coronata* from Nemouridae nymphs **22** allantoid trichospores and accompanying cells **23** zygospores produced homothallically **24, 25***Orphella helicospora* from Leuctridae nymphs: **24** homothallical zygospores and accompanying cells **25** basal cell and holdfast. Scale bars: 25 μm in all figures.

We found this species associated with *Leuctra hippopus* (Kempny 1899) in two Bulgarian rivers and streams. The specimens examined had the typical characteristics of the species, including the straight trichospores measuring 47–56 × 5–7 µm in our collections, with generative cells 21–26 µm long and a supporting cell 6–8 µm length (Fig. 21). All the characters of trichospores and accompanying cells fit the description of the species (Santamaria and Girbal 1998). Zygospores were not seen on this occasion. This species was described from Catalonia, Spain (Santamaria and Girbal 1998, Valle and Santamaria 2005) and has been reported also from Norway (White and Lichtwardt 2004), France (Valle 2013b) and Italy (Valle et al. 2014).

#### Orphella coronata

Taxon classificationFungiOrphellalesLegeriomycetaceae

Léger & Gauthier, 1931.

CC557B29-D1E0-57ED-A98E-BE3EBF175D93

Figs 22 , 23

##### Specimens examined.

Site 10: slide BUL–10–4 (zygo.)

##### Notes.

Collections were made from the hindgut of *Protonemura montana* Kimmins nymphs, with a low infestation rate (2%). *Orphella coronata* has been reported from diverse localities in Europe, (e.g. France (Léger and Gauthier 1931, 1932), Norway (White and Lichtwardt 2004), Spain (Valle and Santamaria 2005), Portugal (Valle 2013a) and Italy (Valle et al. 2013)). *Orphella coronata* seems to be the rarest species of the genus in the Bulgarian streams surveyed, although many potential hosts were dissected from the Struma River. Fortunately, the only *Protonemura* infested specimen was carrying various mature thalli of *O. coronata*, so that we could observe trichospores and the typical heterothallically-formed helicoidal zygospores typical for the species, these being very important to discern and identify possible cryptic species (Valle and Santamaria 2005, Valle et al. 2014, White et al. 2018). Bulgarian specimens show the typical thallus with a bifurcate basal cell and allantoid trichospores (Fig. 22), measuring 36–41 × 5.5–6.5 µm in our collections, slightly smaller than previously reported (35–48 × 6–7.5 µm according to Valle & Santamaria 2004). Terminal cell measures 22–25 × 3–3.5 µm. Zygospores (Fig. 23) in our collections measure 26–32 × 6–7 µm, also somewhat smaller than those reported in the description of the zygospores (30–35 × 5–7 µm according to Valle & Santamaria 2004), but likely attributable to intraspecific variation; in fact, just a couple of thalli were found producing sexual spores in our collections and about 5 producing trichospores.

#### Orphella helicospora

Taxon classificationFungiOrphellalesLegeriomycetaceae

Santam & Girbal, 1998.

825E9593-DC69-5125-A439-385D9D118D23

Figs 24 , 25

##### Specimens examined.

site 4: slide BUL-4-1; Site 7: slide BUL-7-6; site 10: slides BUL-10-2, BUL-10-5.

##### Notes.

Species were obtained from the hindgut lining of Leuctridae nymphs (mainly *Leuctra hippopus*). We found several thalli, most of them producing trichospores and one also bearing helicoidal zygospores, formed homothallically, measuring 25–27 × 5.5–6.5 µm, growing on a fusiform zygosporophore measuring 20–23 × 7–8.5 µm, with a 3 µm long supporting cell and a sigmoid or reflexed intermediate cell about 20–24 µm long in the specimens seen (Fig. 24). All the characteristics of spores and accompanying cells fit those of the specimens reported from other localities in Europe, including Spain (Santamaria and Girbal 1998; Valle and Santamaria 2005), Norway (White and Lichtwardt 2004); Italy (Valle et al. 2013). Thallus has the characteristic basal cell (Fig. 25) and lateral subsidiary branches.

### Order Amoebidiales (Mesomycetozoea)

#### Paramoebidium angulatum

Taxon classificationFungiAmoebidialesLegeriomycetaceae

Valle, 2014a.

0BDA55AB-0AF6-53D7-BFF7-000E6BBDC704

Fig. 26

##### Specimens examined.

Site 4: slide BUL-4-2

##### Notes.

This species is characterised by having a thallus bent approximately at a right angle about one-quarter up the thallus from the holdfast; no other described species shares this feature. This species was originally described from the stonefly family Taeniopterygidae. Our specimens were observed in Nemouridae, a different family in the same Order of insects sharing all characteristics with *P. angulatum*, although most individuals were immature, measuring 350–380 × 29–33 µm, with the typical thallus with the right angle bend and discoid acellular holdfast, known previously only from France (Valle 2014a).

#### Paramoebidium chattoni

Taxon classificationFungiAmoebidialesLegeriomycetaceae

Léger & Duboscq ex LG Valle, 2014b

D5790869-A23B-5C04-BED0-84DC09839F86

Fig. 27

##### Specimens examined.

Site 2: slide BUL–2–6.

##### Notes.

This species of *Paramoebidium* is common within Simuliidae hosts and is identifiable by having the widest diameter of thallus at the basal to middle sections, slightly tapering towards the distal end. It has a non-cellular holdfast located at the proximal end smaller in diameter (12–27 µm) than the thallus, cylindrical or slightly campanulate (Valle 2014b). Bulgarian species perfectly match with the original description (Léger and Duboscq 1948) and with that provided in the validation of the species by Valle (2014b). The species was recently reported from Colombia (Barón and Valle 2018). In our collections, *Simuliomyces microsporus* was often found attached to the thalli of this *Paramoebidium* (Fig. 27).

#### Paramoebidium curvum

Taxon classificationFungiAmoebidialesLegeriomycetaceae

Lichtw., 1979.

4F19093E-64FC-53E2-B39A-D4E8A4579F13

Fig. 28

##### Specimens examined.

Site 2: slides BUL–2–1; BUL–2–6, BUL–2–7, BUL–2–10; Site 12: slide BUL–12–5.

##### Notes.

This species was found attached to the posterior hindgut or anal gills of larval Simuliidae hosts, where we also found *P. chattoni*. In Bulgaria, the species was quite common in the black fly hosts dissected. All the individuals had the typical characteristics of the species (Dang and Lichtwardt 1979), with a holdfast placed on the incurved section of the thallus. This species is known from USA (Dang and Lichtwardt 1979), Sweden (Lichtwardt 1984), Armenia (Nelder et al. 2005), Canada (Strongman and White 2008), Spain (Valle and Santamaria 2009, Valle 2014b), Italy (Valle et al. 2014). *P. curvum* is probably very common and cosmopolitan as their hosts, but their thalli can be easily overlooked when attached to or near to the anal gills.

#### Paramoebidium hamatum

Taxon classificationFungiAmoebidialesLegeriomycetaceae

Bench & MM White, 2012

20C2828D-4BB4-55EB-B502-67E72FAAF838

Fig. 29

##### Specimens examined.

Site 1: slides BUL–1–1, BUL–1–2.

##### Notes.

*Paramoebidium hamatum* was described originally from USA in Chironomidae, Ameletidae and Baetidae (Ephemeroptera). In Bulgaria, it is associated with Baetidae nymphs (*Baetis* sp. *B. rhodani* and *B. melanonyx*). The species was recorded before in Europe (Spain, Busquets et al. 2018). Bulgarian specimens measured 180–300 × 12–25 µm, with the broader diameter at the proximal end, near the holdfast, thinner at the distal end (Fig. 29); cystospores observed, measuring about 10 × 4–4.5 µm. The species was identified by the curved portion at the basal one-eighth to one-third of the thallial length and by its holdfast characteristics (Bench and White 2012).

#### Paramoebidium inflexum

Taxon classificationFungiAmoebidialesLegeriomycetaceae

Léger & Duboscq, 1929

3B0176BE-26ED-55C5-94A8-BB92E340FF75

Fig. 30

##### Specimens examined.

Site 4: slide BUL–4–6.

##### Notes.

Species observed attached to the hindgut lining of *Protonemura montana*, measuring 280–340 × 40–60 µm in our collections. This species was described from France, associated with *Nemoura variegata* nymphs, in the stonefly family Nemouridae. The species has three different thallial morphologies (Léger and Duboscq 1929b, Duboscq et al. 1948). Subsequent to the revision of the species by Duboscq et al. (1948), this endobiont was not reported again, until now, probably because of the thallus variability and, thus, relatively difficult identification. We only saw one of the thallial morphologies described for the species, this being the stouter and shorter thallus type. The other two morphological types show longer and incurved thalli, one type being wider than the other (Duboscq et al. 1948). Neither was observed in our collections.

**Figures 26–30.**
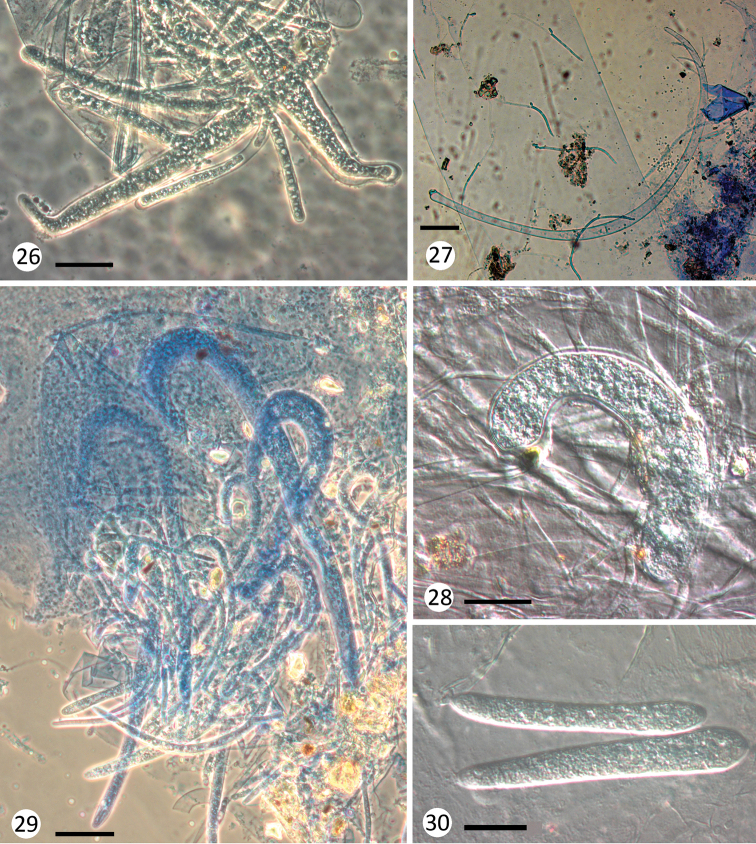
Various species of Amoebidiales. **26***Paramoebidium angulatum* from Nemouridae nymphs, thalli overview with typical angular shape at the upper section **27***Paramoebidium chattoni* from Simuliidae larva, overview, with thin filaments of *Simuliomyces microsporus***28***Paramoebidium curvum* from Simuliidae larva, overview **29***Paramoebidium hamatum* from Baetidae nymphs, overview of various thalli in different growth phases **30***Paramoebidium inflexum* from Nemouridae nymphs, thallus overview. Scale bars: 50 μm (**26, 29, 30**), 100 μm (**27**), 25 μm (**28**).

## Discussion

The new species, *Glotzia balkanensis*, was the most remarkable taxon recorded in this study. As noted above, it is morphologically close to *G. centroptili* (Gauthier ex Manier and Lichtw.), recorded from the western Mediterranean (Gauthier 1936, Busquets et al. 2018) and to *G. distorta* from Italy (Valle et al. 2014), both species from *Centroptilum* hosts. The new Bulgarian species is justified on the basis of spore and thallial characteristics and was found associated with a different host, *Baetis melanonyx*, in two distant sites in the Province of Sofia. Both sites were permanent non-tidal, smooth-flowing watercourses (EUNIS habitat type code C2.3, see Table 1). Moreover, both localities, where the new species was found, had in common a water pH of 7.2, the highest of all the watercourses we surveyed in Bulgaria, but different water temperatures (see Table 1). Another species of the genus *Glotzia* with similar characteristics is *Glotzia stenospora* MM White & Lichtw., from Norway. However this species has larger trichospores measuring 60–68 × 3–5 µm, with a higher length/width ratio (White and Lichtwardt 2004). Additionally, *G. stenospora* has larger zygospores than *G. balkanensis* (61–72 × 11–14 µm, according to White and Lichtwardt 2004). The other non-European species of *Glotzia* have smaller zygospores and most of them (except *G. coloradense* William & Lichtw.) also have smaller trichospores (Lichtwardt et al. 2001).

We gathered other Harpellid species from the guts of Baetidae nymphs, including *Graminella bulbosa*, *Legeriomyces ramosus* and *Spartiella barbata* and the Amoebidiales*P. hamatum*. The species *Graminella bulbosa* has been reported from western Europe, in France (Léger and Gauthier 1937, Manier 1962a), Spain (Valle 2003, 2007), Portugal (Valle 2013a) and Italy (Valle et al. 2014), with this being the first report in eastern Europe. The characteristics of the Bulgarian specimens are interesting indeed, since trichospore morphology is intermediate between two species: *G. bulbosa* and *G. microspora*. We have assigned the specimens to *G. bulbosa*, the type species, which probably is variable enough to actually include both species, as suggested by Valle (2004). Trichospores of *G. microspora* measure 6–8.5 x 2–2.5 μm, according to Lichtwardt and Moss (1984), while trichospores of Bulgarian *G. bulbosa* measure 8–11 × 2 μm. According to Léger and Gauthier (1937) and later validated by Manier (1962a, 1970), trichospores of French *G. bulbosa* measure 9–17 × 2–3 μm. Bulgarian specimens, like many other specimens collected by one of the authors (LGV) in diverse European localities, overlap the measurements of both species. This question will be addressed further in an upcoming paper. *Graminella* is very peculiar amongst Harpellales for its vegetative propagules, originated at, or near, the basal cell. Bulgarian specimens of *G. bulbosa* also had these swollen basal cells, which can extrude their cellular contents to act as vegetative propagules within the same gut. Our specimens were associated with *Baetis rhodani* (Pictet) and *B. alpinus*, both common hosts previously reported with this fungus.

*Legeriomyces ramosus* is a common endosymbiont of Baetidae nymphs and has a broad distribution, especially in the Northern Hemisphere (Lichtwardt et al. 2001). The Bulgarian specimens had the characteristic attributes of the species, including trichospore and zygospore morphology and thallial features. *Spartiella barbata* is often seen together with *L. ramosus*, both sharing the same host, as observed in some of our collections, although *S. barbata* is not as prevalent as *L. ramosus*. The trichospores of *S. barbata* are somewhat similar to those of *L. ramosus*, but more ovoidal and have a single appendage initially folded showing a knob at the proximal end (Manier 1968, Valle 2007). The basal cell of *S. barbata* is also characteristic, with bulbous swellings around the zone of attachment to the host cuticle, a feature not present in *L. ramosus* (Manier 1968, Valle and Santamaria 2002). *Spartiella barbata* has a noticeable preference for calcareous watercourses, but in this study, even though calcareous rocks were present on the river substrate, as in collection site 10 (see Table 1), the water where *S. barbata* specimens were collected, had a slightly acidic pH (6.8 in site 7 and 6.1 in site 10).

Amongst the Amoebidiales inhabiting Ephemeropteran nymphs, we collected and recognised *P. hamatum* from Baetidae hosts. This species described from America (Bench and White 2012) was recorded before from Europe in Italy (Valle et al. 2014) and then Spain (Busquets et al. 2018). Léger and Duboscq (1929b), in a paper dealing with *Paramoebidium*, named *P. arcuatum* from Baetidae nymphs, without providing additional information on the species (no description or illustrations). Subsequently, Duboscq et al. (1948) provided more information on this species, including the identity of Baetidae hosts where it was observed, a description and some line drawings (Duboscq et al. 1948). The characteristics of this species, described from France earlier, are like those of *P. hamatum* and, consequently, very similar to the specimens we have collected within Baetidae hosts in Bulgaria. Probably, *P. hamatum* is the same species named *P. arcuatum* (regarded as *nomen nudum* by Lichtwardt et al. 2001), but further investigation is needed to resolve this question. There are some other *Paramoebidium* species that were named by French authors at the first half of the 20th century, but were not validated or lacked a complete description, making a clear, conclusive identification very difficult.

Other species of *Paramoebidium* were observed from different Ephemeropteran families, including Leptophlebiidae (with some specimens resembling *P. hamatum*), Caenidae and Heptageniidae, but were not identified for the lack of enough material or mature specimens for study. These mayfly nymphs did not have associated Harpellales.

The examined Bulgarian Simuliidae (Diptera) held various species of Harpellales and Amoebidiales. Larval black flies inhabit a wide range of flowing waters, from the smallest streams to the largest rivers (Nelder et al. 2006) and have been collected in nearly all surveyed watercourses with high enough flow velocity (not in lentic and slow moving watercourses). Amongst the trichomycetes they had in our collections, *Genistellospora homothallica* was the most prevalent in the hindgut, occasionally accompanied by *Simuliomyces microsporus*, a smaller species growing epithallically on the robust structure of *G. homothallica*. On the other hand, *Harpella melusinae* was the most common in the mid-gut, attached to the chitinous peritrophic matrix. *Harpella melusinae* is unbranched, placed within the family Harpellaceae and easily identifiable by the coiled trichospores in a series of long generative cells (Lichtwardt et al. 2001). *Stipella vigilans* is not as common as *G. homothallica*, but both species share the hindgut of Simuliidae. The former species can be identified by the mucilaginous substance embedding the basal cell and type I zygospores, according to zygospore types designated by Moss (1975).

In the hindgut, near the anal gills or attached to them, *Paramoebidium curvum* appears also commonly in Simuliidae, this being identifiable by its curved and robust sac-like thallus with a prominent and eccentric holdfast (Dang and Lichtwardt 1979). Another *Paramoebidium* observed within Simuliidae hindguts in Bulgaria was *P. chattoni*, with a longer, arched thallus. Both species of *Paramoebidium* are quite common in various Simuliidae species and have been reported from different countries (Lichtwardt et al. 2001, Valle 2014b). We also collected symbionts of Chironomidae larvae, but just two species: *Stachylina nana* from the mid-gut and *Smittium dipterorum* from the hindgut, both in *Chironomus* larvae. *Smittium dipterorum* was described from Costa Rica (Lichtwardt 1997) and then reported from the Dominican Republic and Mexico (Valle and Cafaro 2010, Valle et al. 2011). It was also recorded from Europe, in Spain (Valle 2007) and it is probably a widespread species, like *Stachylina nana*, but more rare (less prevalent), especially in Europe. There are insufficient data to determine whether this is or is not a cosmopolitan species.

The genus *Orphella* (Orphellales) is associated with Plecopteran nymphs, a host also present in our collections, especially in pristine, high altitude or mountain watercourses, since stoneflies nymphs are very susceptible to water pollution and also water temperature, preferring cold and clean streams and rivers with running water and aquatic vegetation and often pebble stones (Berthélemy 1963). We gathered three species in the Orphellales: *O. coronata* within the hindgut of *Protonemura montana* was the rarest of the *Orphella* species in Bulgaria; however, we were very lucky to obtain several thalli producing trichospores and zygospores from heterothallic conjugations. *Orphella coronata* is the only European *Orphella* with heterothallic sexual reproduction. Only the related North American counterparts with allantoid trichospores, *O. haysii* Lichtw. & Williams (USA) and *O. dalhousiensis* Strongman & MM White (Canada) show the same sexual behaviour (White et al. 2018). The Bulgarian *O. coronata* had slightly smaller trichospores and zygospores than reported before, but differences are not significant; all other characters of accompanying cells and thallial structure matched the description of the species, taking into consideration all recorded geographic variability (White et al. 2018). *Orphella catalaunica* was associated with *Leuctra hippopus* nymphs and most of the observed thalli were immature, except for a couple producing trichospores. No zygospores were observed, but the straight cylindrical trichospores, accompanying cells in fertile cap and thallial characteristics of Bulgarian specimens, fit with those of the original description and later records (Santamaria and Girbal 1998, Valle 2004, White et al. 2018). *Orphella helicospora* was also associated with Leuctridae hosts, in the same species as *O. catalaunica*, sometimes sharing the same gut, which is common for these species. In the case of *O. helicospora*, both trichospores and zygospores were observed and were in accordance with previous descriptions of the species, which has been reported before from various western and central European countries (Lichtwardt et al. 2001). Two species of *Paramoebidium* were also identified within Plecopteran hosts. *P. angulatum*, described from France in Taeniopterygidae has been documented here from a different plecopteran family, the second record of the species. On the other hand, *P. inflexum*, is a rare species described from France that has not been documented until now since Dubosq et al. (1948), in both occasions from Nemouridae nymphs. This is an interesting addition and probably further studies and surveys will allow the possibility of gathering the three different thallial structures described for *P. inflexum*, since only one was observed in our Bulgarian collections.

## Conclusions

This short survey provided 18 new taxa for both Bulgaria and the Balkan Peninsula, including one new species, *Glotzia balkanensis*, with sporic features that allow a clear differentiation from other described species of the genus and which also shares characteristics with the other two European species. The morphological characteristics of *Graminella bulbosa*, collected in Bulgaria, have intermediate spore and thallial characteristics between those of *G. microspora* and *G. bulbosa*, as previously reported by Valle (2004), making mandatory a revision of the European species of the genus. Some rare or poorly known taxa were recovered, including *Paramoebidium inflexum* and *P. angulatum*. Amongst the Orphellales, three species have been reported from Bulgaria (*O. catalaunica*, *O. coronata*, *O. helicospora*). They seem to have a broad distribution in Europe, from the western Mediterranean to the Balkans. Some other taxa, reported here, have a cosmopolitan distribution, such as those associated with Simuliidae larvae, including *Harpella melusinae*, *Genistellospora homothallica* and *Simuliomyces microsporus*. The species associated with the dipteran Chironomidae, *Stachylina nana* have a wide geographic distribution and that is possibly also true for *Smittium dipterorum*; unfortunately, it seems to be less prevalent and there are few reports for this species described in Costa Rica. Our findings support previous observations, revealing that dipteran hosts generally bear endosymbiont species more widely distributed than other insect groups. This may be related to the possibility of adult-mediated transport of fungal diaspores (Moss and Descals 1986, Labeyrie et al. 1996) and also the more restricted flight and dispersal capacity of stonefly and mayfly adults. Amongst Plecopteran hosts, there seems to be a more manifest species delimitation between the Old and New world, as in the case of *Orphella* (White et al. 2018) and also between the North and South Hemispheres. Similarly, the geographic distribution of Ephemeropteran endobionts may be important, but not so evident, as in Plecopteran-associated endobionts. However, these are general tendencies and much more effort has to be spent to improve our knowledge of this poorly-known group of cryptic organisms, regarding diversity, ecology, biology and biogeography. It is our aim to increase the knowledge of trichomycetes in this very interesting biogeographic region.

## Supplementary Material

XML Treatment for Genistellospora homothallica

XML Treatment for Glotzia balkanensis

XML Treatment for Graminella bulbosa

XML Treatment for Harpella melusinae

XML Treatment for Legeriomyces ramosus

XML Treatment for Simuliomyces microsporus

XML Treatment for Smittium dipterorum

XML Treatment for Spartiella barbata

XML Treatment for Stachylina nana

XML Treatment for Stipella vigilans

XML Treatment for Orphella catalaunica

XML Treatment for Orphella coronata

XML Treatment for Orphella helicospora

XML Treatment for Paramoebidium angulatum

XML Treatment for Paramoebidium chattoni

XML Treatment for Paramoebidium curvum

XML Treatment for Paramoebidium hamatum

XML Treatment for Paramoebidium inflexum
